# Stem Cell Transplantation for Neuroprotection in Stroke

**DOI:** 10.3390/brainsci3010239

**Published:** 2013-03-07

**Authors:** Kazutaka Shinozuka, Travis Dailey, Naoki Tajiri, Hiroto Ishikawa, Yuji Kaneko, Cesar V. Borlongan

**Affiliations:** Center of Excellence for Aging & Brain Repair, Department of Neurosurgery and Brain Repair, University of South Florida College of Medicine, 12901 Bruce B. Downs Blvd., Tampa, FL 33612, USA; E-Mails: kshinozu@health.usf.edu (K.S.); tdailey@health.usf.edu (T.D.); ntajiri@health.usf.edu (N.T.); hishikaw@health.usf.edu (H.I.); ykaneko@health.usf.edu (Y.K.)

**Keywords:** stem cells, stroke, cerebral ischemia, transplantation

## Abstract

Stem cell-based therapies for stroke have expanded substantially over the last decade. The diversity of embryonic and adult tissue sources provides researchers with the ability to harvest an ample supply of stem cells. However, the optimal conditions of stem cell use are still being determined. Along this line of the need for optimization studies, we discuss studies that demonstrate effective dose, timing, and route of stem cells. We recognize that stem cell derivations also provide uniquely individual difficulties and limitations in their therapeutic applications. This review will outline the current knowledge, including benefits and challenges, of the many current sources of stem cells for stroke therapy.

## 1. An Overview of Tailoring Stem Cell Therapy for Stroke

Different tissue-derived adult stem cells can be employed as donor cells for transplantation therapy in stroke. An important factor in considering stem cells for therapy is their use as autologous *versus* allogeneic cells. Autologous stem cell treatment involves procuring the cells from the same individual in which the cells will be used, compared to receiving cells from an unrelated donor in the case of allogeneic stem cell transplantation. A potential limitation of allogeneic stem cell grafts includes their predisposition for eliciting an immunogenic complication from the host, such as graft rejection. On the other hand, autologous stem cell transplantation also has limitations. The current research for favorable outcomes suggests an optimal combination of intravenous administration, 48 h post-stroke, and a therapeutic dose of 1 million cells [1,2,3]. This short period of opportunity poses a challenge in generating an ample supply of enough stem cells from freshly harvested autologous tissue sources. Ease of harvesting also has a great influence over the practicality of therapeutic potential, regardless of autologous or allogenic cells. Some of the techniques require highly invasive procedures or present ethical problems with acquiring the stem cells, such as neural stem cells and embryonic stem cells, respectively.

Immunological reactions, such as graft *vs*. host, along with complications secondary to adjunctive immunosuppression, impart another barrier of stem cell treatment for stroke. The immunosuppressant cyclosporine A promotes endogenous neural stem cell activity and migration, thus aiding in the recovery of cortical injury following a stroke [4]. Stroke research in immunocompromised animals has documented elevated endogenous neurogenesis via a CD4^+^ T cell, but not a CD25^+^ T cell-dependent mechanism [5]. Despite the possibility for stem cells to produce an immunogenic response, it is evident that the more naive or less lineage-specific a cell, the less likely it is to invoke an immune response. For example, due to immunological immaturity, umbilical cord blood transplantation is less likely to require immunosuppression. Subsequently, human leukocyte antigen (HLA) matching is less strict preceding transplantation while cell viability remains high compared to the requirements for bone marrow transplants [6]. Mesenchymal stem cells, which can be harvested from a variety of mesenchymal tissues, have different characteristics in an immune response depending on its origin. For example, chorionic plate-derived mesenchymal stem cells show higher expression of HLA-G, which is a contributing factor to induce a stronger immunosuppression [7] and a prognostic indicator of graft tolerance [8], in comparison with bone marrow-derived and adipose tissue-derived mesenchymal stem cells [9]. Placenta-derived mesenchymal stem cells show less inhibition of CD4^+^ T cell stimulation than bone marrow-derived stem cells [10].

In this review, we will provide insights on the different tissues used to harvest stem cells, along with both their limitations and advantages, for stroke neuroprotection. An overview of stem cells currently being investigated for neurorestoration has previously been published [11].

## 2. Embryonic Stem Cells

Embryonic stem (ES) cells have arguably been used as the yardstick of “stemness” properties, providing access to an indefinite supply of stem cells that populate all three germ layers. Two major caveats that hinder the use of ES cells for transplantation therapy relate to the ethical concerns and risk of tumorgenicity [12,13]. In this section, ES cell-derived cell transplantation is discussed and not direct transplantation of ES cells *per se*. ES cell-derived neural progenitor cells transplanted in stroke mice models have been shown to contribute to the repair of neuronal damage [14]. Transplantation of endothelial cells and mural cells derived from ES cells have the potential to contribute to therapeutic vascular regeneration and subsequent reduction of infarct area after stroke in mice [15]. With specific manipulations, human ES cells can be differentiated into neural stem cells called SD56, which do not form tumors after transplantation [16]. Additionally, gene manipulation of ES cell-derived cells have been reported to facilitate therapeutic effects by overexpressing neuroprotective factors such as Bcl-2, adenosine, and myocyte enhancer factor 2C [17,18,19]. Such transplantation of ES cell-derived cells also results in functional recovery in animal ischemic models [17,18,20,21,22].

## 3. Adult Stem Cells

A primary challenge with sources for adult stem cells is the purification of a homogeneous stem cell population, since the adult tissue source contains non-stem cells that have already been committed to specific lineages. The nature of stroke and stem cell mode of action are both diverse. Because of this, there is special consideration given to the use of specific stem cell derivatives to treat specific stroke conditions.

### 3.1. Bone Marrow-Derived Stem Cells

Diverse populations of cells constitute the bone marrow. These cells are purified to harvest an isolated cell type or used as a mixture. Research emerging within the last decade suggests the feasibility of bone marrow-derived stem cells for stroke therapy. Data demonstrate that, upon injury, bone marrow-derived stem cells can mobilize from the bone marrow (BM) and migrate into the peripheral blood. Once in systemic vasculature, they then can enter the central nervous system to influence neuronal injury [23]. Cell constituents of bone marrow include: hematopoietic stem cells (HSCs), mesenchymal stem cells (MSCs), endothelial progenitor cells (EPCs), and very small embryonic-like stem cells (VSELs) [24]. We will outline the therapeutic potential of these bone marrow-derived stem cell lines in the following sections.

#### 3.1.1. Hematopoietic Stem Cells

Hematopoietic stem cells (HSCs), of the phenotype CD34^+^ [25] with additional surface markers CD150^+^, CD244^−^, and CD48^−^ [26], can differentiate into all blood cells. In response to a cerebrovascular incident such as stroke, the CNS can produce cytokines that induce HSC mobilization [27,28,29,30]. Neurotransmitters, most notably catecholamines, can induce HSC mobilization through a nerve ending paracrine signal directly into bone marrow or through sympathetic release into blood circulation [31]. Current treatment protocols, such as granulocyte-colony stimulating factor (G-CSF), apply this cytokine-mediated recruitment [29,30]. Human clinical data of acute stroke shows an abundant mobilization of peripheral blood immature hematopoietic CD34^+^ cells, colony-forming cells, and long-term culture-initiating cells [32]. The magnitude of mobilization appears to correlate with the recovery of function [33]. More so, the infusion of autologous bone marrow mononuclear cells containing HSCs, in addition to cell types including mesenchymal stromal cells and lymphocytes, has been reported in human stroke patients [34,35,36,37]. Those studies, which include the acute, subacute, and chronic phases of stroke, show no adverse effects of transplantation. Transplantation of bone marrow mononuclear cells increases plasma β-nerve growth factor [34]. The amount of CD34^+^ cells in mononuclear cells transplanted shows a trend of positive correlation with rate of functional recovery [34].

#### 3.1.2. Mesenchymal Stromal Cells

Mesenchymal stromal cells, of the phenotype SH2^+^, SH3^+^, SH4^+^, CD29^+^, CD44^+^, CD14^−^, CD34^−^, and CD45^−^[38], are found in nearly all tissues of the body and can differentiated into mesenchymal tissues such as osteogenic, chondrogenic, and adipogenic cells. In this section, we will first discuss the use of mesenchymal stromal cells derived from bone marrow (BM) for the treatment of stroke, but also describe the use of the non-bone-marrow-derived mesenchymal stromal cells.

The use of BM-derived mesenchymal stromal cells prompts functional recovery of neurological deficits succeeding cerebral ischemia in stroke models [39,40,41]. Mesenchymal stromal cell transplantation benefits are imparted by the introduction of neurotrophic factors that activate endogenous brain tissue. These factors include: hepatocyte growth factor (HGF) [39,42], vascular endothelial growth factor (VEGF) [43], nerve growth factor (NGF) [44], brain-derived neurotrophic factor (BDNF) [44], basic fibroblast growth factor (bFGF, FGF-2) [43], and insulin growth factor-1 (IGF-1) [45]. In addition to secreting such factors, the presence of MSCs may promote endogenous induction and migration of primary stem cells from their usual locations (SVZ and SGZ) to the location of injury, while also reducing apoptosis in the penumbral zone of the lesion [43,44]. Whether or not mesenchymal stromal cells differentiate into functional neurons, there is an indication that these cells promote neurogenesis after a stroke injury, but do not have long-term survival after transplantation [43]. Much like neural stem cells, the benefits may also arise from the production of neurotrophic factors such as BDNF, β-NGF [46], and the modulation of vasculature observed equally from four different sources: bone marrow, adipose tissue, skeletal muscle, and myocardium [47]. A clinical trial of intravenous infusion of autologous BM-derived mesenchymal stromal cells in ischemic stroke patients shows significant functional improvement in infused patients without adverse effects in comparison with non-infused patients [48]. Five-year follow-ups of mesenchymal stromal cell-infused patients also show a higher survival rate and functional improvement than non-infused patients [49].

Mesenchymal stromal cells are the most commonly studied stem cell derived from extraembryonic tissue. Unlike neural stem cells from the ectoderm-derived tissue of the nervous system, mesoderm-derived mesenchymal stromal cells can be isolated from nearly all mesenchymal tissues of the body, including bone marrow, placenta, teeth, and adipose tissue. The abundance of potential harvesting sites makes mesenchymal stromal cells a favorable line for autologous transplantation. However, potential discrepancies have prompted the International Society for Cellular Therapy (ISCT) to define minimal criteria for definition of a stem cell as a mesenchymal stromal cell. Plastic adherence, cluster of differentiation (CD) expression, and differentiation ability are some of the characteristics considered [50].

Although mesenchymal stromal cells are harvested from mesenchyme-derived tissues, evidence reports that mesenchymal stromal cells from different locations may impart specific roles as a function of the various ways they are extracted, isolated, and proliferated [51,52,53,54,55]. To this extent, one site of tissue-derived mesenchymal stromal cells may be better qualified for a specific therapy than cells derived from another. Differences also exist between mesenchyme-derived stromal cells and other stem cells. For example, research in bone marrow-derived stem cells in horses established that these cells reach senesce at earlier passages than adipose and umbilical cord-derived cells in mesenchymal tissue [56].

Much like neural stem cells, described later in this paper, the risk of mesenchymal stem cells developing into tumors must be considered. The literature notes a sarcoma developed in the lungs after mesenchymal stem cells were transplanted in mice [57]. Also, secretions from mesenchymal stem cells affect tumors. The combination of interleukin-6 (IL-6) and vascular endothelial growth factor A (VEGF) secreted from mesenchymal stem cells increases the ability of breast cancer cell lines to migrate [58]. Breast cancer cells stimulate *de novo* secretion of the chemokine CCL5 from mesenchymal stem cells, which then acts in a paracrine fashion on the cancer cells to enhance their motility, invasion, and metastasis [59]. Consequently, mesenchymal stem cells of specific derivations may have a greater propensity for tumorigenesis and encouraging metastasis. This may not be the case for all mesenchyme-derived stromal cells, however. Research suggests umbilical cord mesenchymal stem cells do not appear to develop into tumor progenitor cells in the presence of tumor cells, unlike bone marrow-derived mesenchymal stromal cells [60].

#### 3.1.3. Endothelial Progenitor Cells

Stroke is multifactorial in etiology. One such factor involves the disruption in vascular integrity, causing vessel vulnerability that predisposes the region to a stroke-like event. The endothelium modulates the permeability of the blood-brain-barrier and thus stroke recovery. Endothelial progenitor cells (EPCs) are precursors for the mature endothelium that lines the vascular system, a role that has long been established [61]. EPCs are defined as cells that express HSC markers such as CD34 or CD133 and the marker protein vascular endothelial growth factor receptor 2 (VEGRF2) [62]. In an early study, transplanted EPCs were found in newly vascularized endothelium of surgically induced ischemic hind limb injury in rabbits [63]. More recent research indicates that circulating BM-derived EPCs are signaled to sites for neovascularization, where they will differentiate into endothelial cells [64,65]. A correlational study in human ischemic stroke patients indicates that the level of circulating EPCs relates to improvement on the National Institute of Health Stroke Scale [66]. An animal model of stroke shows that tail vein injection of EPCs reduces infarct induction through middle cerebral artery occlusion (MCAO) in diabetic mice [67]. Also, intravenous infusion of autologous EPCs after MCAO in rabbits shows functional improvement, decreasing the number of apoptotic cells, increasing microvessel density in the ischemic boundary area, and diminishing the infarct area [68]. The research of EPCs and stroke-related vascularization is still sparse, but the evidence is surmounting that they could play a constitutional role in the prevention of stroke and the treatment after an injury.

#### 3.1.4. Very Small Embryonic-Like Stem Cells

Much like the hematopoietic stem cells discussed above, very small embryonic-like stem cells (VSELs), which have the phenotype Sca-1^+^, lin^−^, CD45^-^ and also have pluripotent stem cell markers such as SSEA-1, Oct-4, Nanog, and Rex-1 [69], are mobilized from adult tissues into the peripheral blood following a stroke event [70,71,72]. The current hypothesis is that VSELs are epiblast-derived pluripotent stem cells that are deposited early during embryonic development [73,74], serving as a reserve within the tissue that can be utilized for rejuvenation. The brain is one such location that includes a large number of cells displaying the VSEL phenotype [75,76]. The ability for VSELs to differentiate into neurons, oligodendrocytes, and microglia to regenerate damaged CNS makes them an excellent candidate for stroke therapy [23]. However, limitations currently exist when considering the use of VSELs. One such obstacle is the low yield of VSELs from harvesting. This restraint requires the necessity for proliferation prior to transplantation [23]. Another restriction is the decrease in number of VSELs with age, thereby exacerbating the difficulty in harvesting an adequate number of cells in older individuals [77].

### 3.2. Neural Stem Cells

In terms of stroke injury, the use of neural stem cells (NSCs) seems like an apparent solution. Endogenous stem cells are located in the subgranular zone (SGZ) of the dentate gyrus, the subventricular zone (SVZ), and the subependymal zone (SEZ) of the spinal cord. As one may anticipate, the cellular activity is upregulated in these zones following a stroke-like injury; yet, this action does not provide cell replacement or full functional repair, despite NSCs being found at the site of ischemic lesions from day 1 after stroke in human patients [78].

NSCs in the SVZ migrate into ischemic lesions after stroke. NSCs from the SVZ are redirected from their normal route through the rostral stream into a redefined direction to reach ischemic regions along blood vessels as a scaffold for migration [79,80,81]. Chemokine signals such as stromal-derived factor-1 (SDF-1), vascular endothelial growth factor (VEGF), and angiopoietin are released from ischemic tissue, influencing the course of the SVZ NSCs toward a path along blood vessels to reach the infarcted area [82,83,84,85]. In *ex vivo* cultures of rat brain cells, microglia from ischemic brain, but not from intact brain, promotes differentiation of SVZ NSCs into neurons, suggesting that microglia might have a role on differentiation of NPCs [86]. However, *in vivo* studies demonstrated that a very low number—or even possibly none of the newborn cells—develop into mature neurons [87,88,89].

While endogenous NPCs migrate and differentiate into mature neurons, this may not be sufficient for self-repair of ischemic brain. Current literature explores the idea of exogenous stem cell transplantation eliciting endogenous stem cell production at the site of injury [90,91]. The intravenous infusion of neural progenitor cells produces increased dendritic length and an increased number of branch points in host neurons [92]. Transplanted NSCs have therapeutic effects without differentiating into mature neurons [93], although there is a report that transplanted ES cell-derived NSCs in the ischemic rat brain differentiated into neurons, into oligodendlocytes in stroke regions undergoing remyelination, and into astrocytes extending processes toward stroke-damaged vasculatures [94]. Even with the beneficial effects of NSC transplantation on endogenous stem cell proliferation, it still has limitations. A primary limitation is the acquisition of these cells. An autologous treatment would require invasive surgery prior to therapy and allogenic grafts would likely require a fetal source or derivation from an alternative cell type. Another possibility would be harvesting the cells during other surgical procedures [95], but this may not be very advantageous. One of the foremost concerning consequences is the potential of stem cells to be tumorigenic. Somewhat contradictorily of immunogenicity, the less differentiated the cell, the greater the potential for the cell line to generate aberrant proliferation. Thus, adult stem cells, due to progressive differentiation, are less likely than embryonic stem cells to encourage tumorigenesis. Additionally, when utilizing stem cells, it is essential to ensure that the transplantation consists of a purified cell population [96,97]. A prior case identified this necessity when a child with ataxia telangiectasia was transplanted with a heterogeneous mixture of fetally derived neural stem cells and was diagnosed with a glioneuroal neoplasm four years later [98]. 

A possible explanation for the potential reduction in tumorigenesis of adult-derived stem cells is due to their reduced capacity to proliferate. A likely benefit for avoiding neoplastic events is, unfortunately, a problem when attempting to achieve a sufficient number of stem cells for transplantation. To navigate these limitations, researchers have developed methods such as long-term culturing, immortalization, insertion of oncogenes, or even deriving neural stem cells from other tissues or from pluripotent stem cells. However, each of the aforementioned methods has inherent limitations. Long-term culturing, for instance, bears the risk of spontaneous conversion to a non-neural cell type, such as a tumor precursor cell [99]. In spite of the teratocarcinoma-derived hNT neuron cell lines advancing into a phase II clinical trial in stroke patients [100], no significant improvements were observed [101]. Oncogene insertion may still have a favorable future. ReNeuron LTD, a stem cell therapeutics company based in England, is using a c-Myc regulator gene and mutated estrogen receptor transgene to generate an immortalized neural cell line [102]. This protocol is currently undergoing clinical trials for stroke in the United Kingdom [103].

### 3.3. Extraembryonic Stem Cells

Tissues rich in extraembryonic stem cells include: umbilical cord, placenta, amnion, and Wharton’s jelly. As discussed above, mesenchymal stromal cells are the most popular cell line for study, but amnioitic epithelial cells, amnion-derived stem cells, placental-derived stem cells, and umbilical cord matrix stem cells can also be found in extraembryonic tissue [104]. Extraembryonic stem cells, much like NSCs and mesenchymal stromal cells, pertain to different germinal layers. The ectoderm gives rise to the amniotic epithelium, while the amnion-derived mesenchymal stromal cells are found in the mesodermal layer [105]. Therefore, amnion-derived stem cells appear to contain a higher capacity for mesodermal cell lineages than the ectoderm [106]. Amnion mesenchymal stromal cells also exhibit less endothelial capabilities, further demonstrating potential embryonic specificity [107]. 

Current studies with extraembryonic stem cells investigate transplanting animal models of stroke with placental-derived mesenchymal stromal cells. In congruence with the proposed mechanism of action, these cells do not appear to solely replace damaged cells. Rather, they appear to furnish the microenvironment in a way that promotes endogenous neurogenesis [108,109,110]. Research with umbilical cord lining mesenchymal stromal cells in rat stroke models demonstrates functional recovery, increased vascular density, increased expression of vascular endothelial growth factor, and basic fibroblast growth factor [111]. Mesenchymal stromal cells derived from the umbilical cord lining also provide an immunosuppressive effect on the immune cascade and appear to have greater immunological immaturity than aged bone marrow mesenchymal stromal cells [112]. Additionally, Wharton’s jelly-derived mesenchymal stromal cells differentiate into glial, neuronal, doublecortin^+^, CXCR4^+^, and vascular endothelial cells to enhance neuroplasticity in the ischemic brain [113] and have an immunosuppressive function by secreting leukemia inhibitory factor (LIF) [114].

The popularity of umbilical cord banking has been increasingly steadily. Given their possibility for both allogenic and autologous use, these stem cells could have broad therapeutic potential. Umbilical cord blood routinely refers to the mononuclear fraction, which includes hematopoietic progenitors, lymphocytes, monocytes, and mecenchymal stromal cells. Even with its heterogeneity, these cells are considered immunologically immature. Thus, these cells have been reported to modulate the immune response and reduce proinflammatory cytokine levels [115]. In terms of transplantation of umbilical cord blood in animal stroke models, there have been auspicious results. Transplantation of umbilical cord blood-derived stem cells in animal models of stroke demonstrates functional recovery, reducing infarct size, and higher expression of neuroprotective factors, such as BDNF and VEGF [1,2,116,117].

### 3.4. Other Sources of Adult Stem Cells

#### 3.4.1. Adipose Tissue

Adipose tissue includes adipose-derived stem cells, which are a plastic-adherent cell population and have a more than 90% identical immunophenotye compared to bone marrow-derived mesenchymal stromal cells [118]. Studies with adipose-derived stem cells exhibited reduced infarct size, improved neurological function, reduced level of cerebral inflammation, and chronic degeneration in an intracerebral hemorrhage model [119,120]. Adipose-derived stem cells can differentiate into neural, glial, and vascular endothelial cells, and also show higher proliferative activity with greater production of VEGF and hepatocyte growth factor in comparison with bone marrow-derived stromal cells [121]. Treatment with adipose-derived stem cells in an ischemic stroke model of mice shows remarkable attenuation of ischemic damage [121].

In spite of the potential benefits for stroke injury as previously noted, there are still side effects associated with adipose-derived stem cells. Extensive passaging of adipose-derived stem cells might cause spontaneous mutations within the cell line that may promote a cancerous state [122]. However, this statement has since been retracted due to the inability to replicate the data [123]. Revisions to these studies have now demonstrated that adipose-derived stem cells can promote preexisting cancerous cells to produce tumors, but do not result in tumors alone [124]. A careful analysis of risk-to-benefit ratio must be observed in order to advance a safe and effective cell therapy for stroke.

#### 3.4.2. Menstrual Blood

With the endometrial lining in the uterus cycling monthly, it has been a location of interest for researchers. Two separate groups have isolated stem cells in this region, although it is unsure if they are the identical cell line due to differences in culturing protocols [125,126]. Menstrual blood-derived stem cells exhibit multipotency. Menstrual blood-derived stem cells secrete trophic factors such as VEGF, BDNF, and NT-3 in response to oxygen glucose deprivation (OGD), an *in vitro* model of stroke. Co-culture of rat primary neurons with menstrual blood-derived stem cells, or its conditioned medium exposed to OGD, improved cell survival rate after OGD [127]. Both intracerebral and intravenous transplantation of menstrual blood-derived cells into stroke model rats improved host cell survival and behavioral functions [127]. These cells have also been implemented for *in vivo* surgical MCAO rat studies without immunosuppression [127,128,129].

#### 3.4.3. Breastmilk

Mammary tissue includes stem cells. Stem cells and differentiated cells from the lactating epithelium enter breastmilk either through cell migration and turnover and/or as a consequence of the mechanical shear forces of breastfeeding [130,131]. Breastmilk stem cells show embryonic stem cell-like morphology and phenotype, and can be differentiated into cell lineages from all three germ layers *in vitro* [131]. The presence of stem cells in the breastmilk may provide a great advantage for harvesting these cells while avoiding any invasive procedures [132]. Historically, the benefits of breast milk have been considered nutritive and immunologic, but emerging research is attempting to elucidate the potential effects of vertical transmission of stem cells from mother to offspring [125]. Accompanying the ease of harvesting, breastmilk stem cells also present the potential for autologous transplantation.

#### 3.4.4. Dental Tissue

Dental tissue could prove to be a useful resource in harvesting stem cells in the future. Dental tissue-derived stem cells such as post-natal dental pulp stem cells (DPSCs) [133], stem cells from exfoliated deciduous teeth (SHED) [128], periodontal ligament stem cells (PDLSCs) [134], stem cells from apical papilla (SCAP) [135,136], and dental follicle precursor cells (DFPCs) [137], which exhibit mesenchymal stromal cells-like capabilities, have been identified (for review, see [138]).

Dental tissue-derived stem cells can differentiate into a variety of cell types including neural cells, adipocytes, and odontoblasts [139]. Transplantation into intact mouse brain showed cell survival along with expression of neuronal markers [139]. A rodent model of cerebral ischemia shows improved sensorimotor function after receiving transplantation of dental tissue-derived stem cells [140,141]. Transplanted DPSCs differentiate into astrocytes in preference to neurons, suggesting secretion of trophic factors for therapeutic effects [141]. Neurogenicity of dental tissue-derived stem cells is more potent than that of bone marrow-derived stem cells [142], most likely due to their neural crest origin [138].

#### 3.4.5. Induced Pluripotent Stem Cells

Once thought to be unidirectional, recent experiments suggest stem cells can be manipulated into their former multipotency. It was originally considered that stem cells progress through maturation to become terminally differentiated. However, the literature indicates that through the transfection of specific transcription factors, embryonic-like stem cells can be regenerated from fibroblasts through retrograde manipulation [143]. This transfection technique has also been applied to umbilical cord, placental mesenchymal stromal cells, neural stem cells, and adipose-derived precursor cells to increase their potency [144,145].

A major benefit of retrograde conversion is the proliferation capacity of precursor cells. Some studies demonstrated beneficial effects of transplantation of induced pluripotent stem cells (iPSCs) in an animal model of stroke, including effects such as: improving sensorimotor functions [146,147], reducing infarct size, reducing pro-inflammatory cytokines, and increasing anti-inflammatory cytokines [146]. However, the use of iPSCs appears to have some ramifications. As with many stem cells, both immunogenicity and tumorigenesis are of concern. The transfection technique used to generate precursor cells utilizes transcription factors of known oncogencity. iPSCs, even when autologous, have also provoked an immune response leading to rejection [148]. In fact, a higher rate of tumorigenesis after transplantation of undifferentiated iPSCs is reported [149,150]. However, pre-differentiated neuroepithelial-like stem cells derived from human fibroblast derived-iPSCs enhances recovery after stroke without forming tumors by four months post-transplantation [151].

In terms of translational research, although iPSCs have the potential for autologous cell therapy, the technology will need to be significantly improved before this becomes a viable option to treat the acute phase of stroke, specifically the demonstration of feasibility that a well-defined population of IPSCs are banked prior to injury due to the duration required to make enough stem cells for a therapeutic dose. Moreover, any genetic manipulation to IPSCs needs to be regulated, particularly in the post-transplantation period, in order to avoid any potential of tumorgenic or ectopic tissue formation.

## 4. New Stem Cell Approaches: Co-Transplantation, Combination Therapy, and Others

As evident by the literature thus far, individual stem cells confer discrete therapeutic potential. Thus there is the potential for treatment with multiple stem cell lines, simultaneously. There is evidence of co-transplantation providing synergistic effects on stem cell survival. One such study demonstrated increased neural stem cell survival when neural stem cell delivery was combined with adipose-derived stem cells [152]. An additional study reports that co-transplantation of bone marrow-derived stromal cells with embryonic stem cells decreased the propensity for tumorigenesis [153]. With similar regard, accompanying neural stem cells with epithelial cells increased survival and differentiation [154].

Combination therapy, similar to co-transplantation of two cell lines, can incorporate a non-stem-cell substrate to increase the efficacy of transplantation. Examples include combining bone marrow-derived stromal cells with trophic factors to enhance survival and potentiation [155] or providing a scaffold for stem cell adherence [156]. The techniques of co-transplantation and combination therapy are still novel, but the ability to enhance stem cell survival while decreasing adverse events is emerging with current research.

Many of the persisting variables in stem cell techniques have recently been reviewed by us [157]. Factors including optimal dose, route of administration, and sex of donor/recipient are all likely to be contingent upon the cell type being investigated. We have investigated many of these factors with umbilical cord blood for conditions such as amyotrophic lateral sclerosis, Alzheimer’s disease, and Sanfilippo syndrome [157]; however, this information has yet to be resolved in regards to stroke. The Stem Cell Therapies as an Emerging Paradigm in Stroke (STEPS) program was designed for the purpose of study interpretation in an attempt to standardize procedures [158,159,160,161].

## 5. Conclusions

As we reviewed here, there is currently a vast number of sources available for stem cell harvesting, and as the evidence is further substantiated, they may each impart their own benefits and have their own native limitations. Many logistical considerations must be made for the use of stem cells for therapeutic stroke treatment. Such factors include mode of action, immunogenicity, tumorigenicity, harvesting, proliferation capacity, and overall feasibility of use. These variables must be addressed before translational studies can proceed. However, despite the limitations identified and the considerations still needing concrete exploration, limited clinical trials of stem cell therapy for stroke patients are already underway. Parallel laboratory investigations are necessary to further optimize the safety and efficacy of stem cells for clinical applications.
