# Addendum: Shinozuka, K. et al. Stem Cell Transplantation for Neuroprotection in Stroke. *Brain Sci.* 2013, *3*, 239–261

**DOI:** 10.3390/brainsci7110145

**Published:** 2017-10-31

**Authors:** Kazutaka Shinozuka, Travis Dailey, Naoki Tajiri, Hiroto Ishikawa, Yuji Kaneko, Cesar V. Borlongan

**Affiliations:** Center of Excellence for Aging & Brain Repair, Department of Neurosurgery and Brain Repair, University of South Florida College of Medicine, 12901 Bruce B. Downs Blvd., Tampa, FL 33612, USA; kshinozu@health.usf.edu (K.S.); tdailey@health.usf.edu (T.D.); ntajiri@health.usf.edu (N.T.); hishikaw@health.usf.edu (H.I.); ykaneko@health.usf.edu (Y.K.)

It has been brought to our attention that the Acknowledgement section is missing in our published paper [1], and therefore we would like to add it as follows:

**Acknowledgments:** C.V.B. is supported by James and Esther King Foundation for Biomedical Research Program 1KG01-33966 and NIH NINDS RO1 1R01NS071956-01.

This addendum does not cause any changes to results and conclusions in the original published paper.
